# Fibrolamellar hepatocellular carcinoma treated with atezolizumab and bevacizumab: two case reports

**DOI:** 10.1186/s13256-021-02695-8

**Published:** 2021-03-16

**Authors:** Ali AL Zahrani, Ali Alfakeeh

**Affiliations:** Department of Medical Oncology, Comprehensive Cancer Center, King Fahad Medical City, Makkah Al Mukarramah Branch Road, Riyadh, 12231 Kingdom of Saudi Arabia

**Keywords:** Fibrolamellar hepatocellular carcinoma, Atezolizumab, Bevacizumab, Immunotherapy

## Abstract

**Background:**

Fibrolamellar hepatocellular carcinoma is a unique tumor of the liver that differs from the classical hepatocellular carcinoma in diagnosis, behavior, and possibly treatment. There is usually absent underlying liver disease, and it usually occurs in young patients. The survival outcomes in localized fibrolamellar hepatocellular carcinoma are perhaps better than in classical hepatocellular carcinoma if treated early and radically. On the other hand, the prognosis remains poor for locally advanced and metastatic fibrolamellar hepatocellular carcinoma. Many reports suggested a limited benefit from systemic chemotherapy. Sorafenib also did not show major effects on fibrolamellar hepatocellular carcinoma. Given the rarity of fibrolamellar hepatocellular carcinoma, lack of large studies, and absence of standard treatment, the treatment decisions rely on case reports, previously reported cases series, and expert opinions. Recent studies have shown promising effects of immunotherapy with checkpoint inhibitors in the first- and second-line therapy of hepatocellular carcinoma. Atezolizumab with bevacizumab regimen has been approved recently as a first-line treatment for classical hepatocellular carcinoma. Currently, there are no reports yet on the use of atezolizumab with bevacizumab for fibrolamellar hepatocellular carcinoma.

**Case report:**

In this article, we present two Arabic patients with advanced fibrolamellar hepatocellular carcinoma who received atezolizumab and bevacizumab combinations but did not show any clinical benefits.

**Conclusion:**

While atezolizumab and bevacizumab combinations had shown benefits in classical hepatocellular carcinoma, the current data showed a lack of benefit and tumor response in fibrolamellar hepatocellular carcinoma.

## Background

Fibrolamellar hepatocellular carcinoma (FLC) is a unique and rare tumor of the liver that was historically classified as a pathological subtype of hepatocellular carcinoma. Edmondson first described it in 1956 in a 14-year-old female with no underlying liver disease [1], and it was later further characterized by Berman *et al*. [2] and Craig *et al*. [3] in the 1980s. The epidemiology, etiology, treatment, and prognosis of FLC differ substantially from classical hepatocellular carcinoma (HCC). FLC is a very rare tumor. In the United States, for example, it is less than 1% of all primary liver tumors [4], while in Mexico, FLC represents 5.8% of all primary liver cancers [5]. FLC usually affects younger individuals. According to data from the Surveillance, Epidemiology, and End Results (SEER) program for FLC cases collected between 1 January 2000 and 31 December 2010, there appear to be two peak incidences: one from age 10 to 30 years and the other between 70 and 79 years [6]. In another SEER database analysis, the average age was 39 years, and 60% of patients were diagnosed under the age of 40 [7]. Usually, there is no underlying cirrhosis or chronic viral hepatitis [8]. Recent advances in molecular studies of FLC found a unique DNAJB1–PRKACA fusion transcript in the majority of cases studied, and this has been linked to possible tumor development in FLC [9, 10]. This fusion is not specific to FLC and has been reported in other malignancies [11]. In a series of 95 patients, the most common symptom was abdominal pain (42%), followed by abdominal mass and distention (24%), and anorexia or weight loss (23%). Jaundice was present in up to 40% of cases [12]. Serum alpha-fetoprotein (AFP) levels are elevated in 10% of cases [13]. Some studies suggest the use of other tumor markers, such as serum des-gamma-carboxy prothrombin (DCP), vitamin B12 binding capacity (B12BC), haptocorrin, and neurotensin [14–16]. The diagnosis is usually made histologically, which usually reveals well-differentiated, large, polygonal tumor cells with eosinophilic hyaline cytoplasmic bodies. These cells are surrounded by fibrous stroma arranged in thin, parallel lamellae [17]. Ultrasound is usually nonspecific and just shows a large mass in the liver.

On computerized tomography (CT) scan they appear as a single large tumor with dense fibrotic bands forming a central scar (seen in approximately 75% of cases). It might be associated with few small calcifications. Sometimes few regional nodal enlargements are seen. Enhancement is usually arterial, and the central scar usually shows persistent enhancement on delayed images.

In magnetic resonance imaging (MRI), the central scar, if present, is usually hypointense on all sequences. Occasionally it may be T2 hyperintense. Because of the lack of fat content no signal is lost on out-of-phase imaging [18].

In a study of 94 cases of FLC, the median survival was 84.9 months for patients with stage I to II (according to the sixth edition of the American Joint Committee on Cancer [AJCC] staging classification from 2002), 54.2 months for stage III (T4 tumor or N1 disease), and 28.9 months for stage IV (distant metastases) [19]. Other studies have indicated that the 5-year survival is 34–70%, which is better than that of classical HCC (10–16%) [6], but the difference is possibly due to the absence of underlying liver diseases in FLC cases [20]. The primary treatment of FLC is surgical resection if localized and surgically resectable, although recurrence rates of up to 90% have been reported. In unresectable FLC, a curative option with transplantation has similar survival rates to transplanted classical HCC [21]. Treatment in cases with metastatic or locally advanced cases beyond transplants options presents a challenge, with no standard recommendations for treatment. Systemic chemotherapy showed some benefits in some FLC patients [12]. In phase II clinical trial, the combination of continuous infusion 5-FU and subcutaneous interferon-α was associated with a response rate of 62% and a median overall survival of 23 months [22]. Some case reports suggest efficacy for platinum-based regimens, such as cisplatin, epirubicin, and fluorouracil (FU) combinations [23, 24]. However, patients treated with systemic chemotherapy alone still have poor prognosis [19]. Targeted therapies such as sorafenib have been used in the treatment of FLC but with limited efficiency. In one study, sorafenib was given to ten patients; after 2.5 to 7 months of treatment, eight had disease progression, one had a mixed response followed by progression, and one was lost to follow-up [12]. The first randomized phase II clinical trial for FLC had three arms: the mTOR inhibitor everolimus, estrogen-deprivation therapy with leuprolide plus letrozole, and everolimus plus estrogen-deprivation therapy. This study was closed early because of a lack of improvement in progression-free survival among the three study arms [25]. One study showed a response of large FLC after using selective internal radiation therapy (SIRT) with Yttrium-90, allowing the patient to undergo curative surgical resection [26]. Recently, an international, open-label, randomized phase 3 trial showed that patients with unresectable hepatocellular carcinoma treated with atezolizumab combined with bevacizumab resulted in better overall and progression-free survival outcomes compared to sorafenib [27]. However, patients with known FLC were excluded from the trial. Here, we report two cases of metastatic FLC who progressed on atezolizumab and bevacizumab combination.

## Case report

We report two cases with fibrolamellar hepatocellular carcinoma that were started on atezolizumab and bevacizumab.

### The first case

A 33-year-old Arabic female was diagnosed with Alagille syndrome during early childhood after she presented with cholestasis. She was managed conservatively, and her disease was stable during the last 10 years. There were no cardiac or renal abnormalities, but there were dysmorphic facies, consisting of a broad nasal bridge, triangular facies, and deep-set eyes. Total bilirubin was 17.5 umol/l (3–20 umol/l), direct bilirubin 13.3 umol/l (0–8.6 umol/l), gamma-glutamyl transferase (GGT) 436 u/l, and alpha-fetoprotein was < 2. During follow-up, she started to complain of abdominal pain and loss of appetite. CT scan in January 2019 showed a liver mass in segment VIII measuring 8 cm × 7 cm with central scar. The lesion was attached to portal vein. For further characterization, MRI had confirmed the findings. A biopsy was consistent with fibrolamellar HCC. She was discussed on the tumor board and was found not resectable. She underwent transarterial chemoembolization (TACE), which resulted in a complete radiological response. In November 2019 she had satellite lesions around the previous one and underwent another session of TACE, which resulted again in a complete radiological response. In March 2020, she was found to have a local recurrence with lung metastases and endoscopy did not show varices. She was discussed on the tumor board; it was felt she would not be able to tolerate combination chemotherapy, and the decision was to start atezolizumab with bevacizumab. She consented and received two cycles, 1200 mg of atezolizumab plus 15 mg/kg of body weight of bevacizumab intravenously every 3 weeks; the last cycle was on 9 June 2020. Cycle 3 was delayed due to elevated transaminases. She was started on steroids without improvement. CT scans showed progression locally and distantly. Her general condition deteriorated further, and she was referred to palliative care.

### The second case

A 22-year-old Arabic male presented with obstructive jaundice symptoms with abdominal mass and loss of weight of 20 kg over the past 3 months. His performance status was 2, he was deeply jaundiced, and there was a palpable liver 12 cm below the costal margin, non-tender with a smooth surface. Total bilirubin was 123 umol/l (3–20 umol/l), direct bilirubin 86 umol/l (0–8.6 umol/l), and alpha-fetoprotein was < 2. MRI showed a large heterogeneous intermediate T2 signal intensity lesion involving all segments of the right hepatic lobe. It measured 20 × 14 × 15 cm. It is associated with the mass effect and obstructs the biliary tree at the level of the porta hepatis. There were also multiple enlarged retroperitoneal lymph nodes. CT of the chest showed multiple bilateral lung nodules and aggressive suspicious bony lesion in the thoracic cage. He underwent ERCP with plastic stent insertion into the hepatic duct and pancreatic duct, with an improvement of biliary obstruction. Bilirubin levels started to decrease, and his general condition improved. Tru-cut biopsy showed fibrolamellar hepatocellular carcinoma. He started on 1200 mg of atezolizumab plus 15 mg/kg of body weight of bevacizumab intravenously every 3 weeks with good tolerance. After two cycles, he started to deteriorate with more obstructive symptoms and signs of progression documented by CT locally and distantly.

## Discussion

FLC is a unique and rare malignancy of the liver that mainly affects younger individuals without underlying chronic liver disease. The prognosis remains poor for patients whose disease cannot be resected completely and for those with extrahepatic disease [12]. Because of the rarity of FLC, there are limited studies for treating advanced disease, with minor advances in recent years [28]. The best treatment approach for those patients is not established, and most of the treatment decisions are either extrapolated from classical HCC or based on reports from small series and case reports. Few small studies show some benefit of different combinations of systemic chemotherapy [28]. Sorafenib, a standard treatment in classical HCC, showed only limited efficiency in FLC [13]. More recently, the finding of the mTOR pathway activation in FLC cells has suggested a potential target for mTOR inhibitors. However, this was not translated into clinical benefits in a randomized clinical trial [4].

Many recent studies established the role of checkpoint inhibitors in several solid malignancies [6] and as first- and second-line therapy in classical HCC [27, 29, 30]. In general, the high mutational burden and tumor's genomic instability are associated with improved overall survival [31]. FLC may have a low mutational burden, which is associated with an inadequate response to immunotherapy. One study reported 41% of MSH2 mutations with next-generation sequencing of FLC tumor tissue. In this study, three cases of metastatic FLC progressed after 2–3 months of starting PD-L1 antibodies, one treated with pembrolizumab, and the other two received nivolumab, although the progression was below 20% [32].

The benefit of bevacizumab was reported in one case report in combination with erlotinib. The case was a metastatic FLC with a previous progression on 5-FU plus interferon-α and FOLFOX regimens. There was a good palliative subjective response with complete resolution of cancer-related pain, with a partial response after 2 months of treatment in distant metastases [33]. Data from the IMbrave150 trial that was published recently showed that the combination of atezolizumab plus bevacizumab improved survival and significantly delayed deterioration in quality of life compared with sorafenib in the treatment of patients with unresectable classical hepatocellular carcinoma (HCC). It showed that atezolizumab plus bevacizumab reduced the mortality risk by 42% compared with sorafenib monotherapy. The median time to deterioration in quality of life was 11.2 months for atezolizumab plus bevacizumab versus 3.6 months with sorafenib. Although serious side effects occurred in as many as 38% of the patients who received the combination therapy, none of the reported side effects were unexpected. Patients with known FLC were excluded from this trial [27]. Both of our cases had failed with the combinations of atezolizumab plus bevacizumab, with evidence of disease progression within 2 months. We are not sure if this failure is due to ineffective treatment or advanced disease. The documented disease progression by radiology may support the ineffectiveness of this combination. However, one might argue that these two patients might be bad candidates or did not have good criteria to receive more courses to show activity. The ineffectiveness and the early progression were also noticed in the study that used immunotherapy in the three cases of metastatic FLC [32].

## Conclusion

To the best of our knowledge, these two cases are the first FLC cases that were treated with atezolizumab and bevacizumab and did not show any meaningful clinical benefits. Further better understanding of FLC is needed, and different treatment options should be assessed through clinical trials.

## Data Availability

All the information and data of both patients are available in the hospital files for both patients
